# Scientific Items

**Published:** 1877-06-20

**Authors:** 


### Scientific Items.

MATERIALISTIC PSYCHOLOGY.—In a recent address delivered before the Victoria Philosophical Institution, reported in the London medical journals Dr. Winn summed up his arguments in the following manner:

1st. It was evident that no great discovery as regards the functions of the brain and nervous system has been made since the days of Sir Charles Bell and Marshall Hall.

2d. That in no instance has it been found that vital and physical force were interchangeable. The doctrine, therefore, of correlation of force could not be applied to the elucidation of vital or mental phenomena.

3d. That respecting the nature of insanity, there are questions which cannot be answered by materialistic psychology, and that a recent attempt to frame a clasification of mental diseases without taking the physical element into consideration had proved an utter failure.

4th. That memory which it had been confidently asserted was a bodily function, could not be localized in any part of the brain, the microscope as yet having failed to detect the faintest indication of the symbols of any language impressed on the cells of the brain.

5th. That the doctrine of unconscious cerebration, of which we have lately heard so much, and which, if true, would reduce man to a mere automaton, admits of an explanation that does not require us to recognize the exclusion of the highest faculties of mind.

